# ASSOCIATIONS BETWEEN COGNITIVE CHANGE AND OBJECTIVE CHANGE IN PHYSICAL ACTIVITY AMONG OLDER ADULTS: THE LIFE STUDY

**DOI:** 10.1093/geroni/igad104.3196

**Published:** 2023-12-21

**Authors:** Erta Cenko, Rachael Seidler, David Cheng, Thomas Pearson, Todd Manini

**Affiliations:** University of Florida, Gainesville, Florida, United States; University of Florida, Gainesville, Florida, United States; University of Florida, Gainesville, Florida, United States; University of Florida, Gainesville, Florida, United States; University of Florida, Gainesville, Florida, United States

## Abstract

**Objective:**

To investigate the associations between domain-specific cognitive change and objectively measured change in physical activity in a sample of community-dwelling older adults. We hypothesized that domain-specific changes in cognition are associated with longitudinal changes in physical activity levels.

**Methods:**

We used data from 955 participants from The LIFE Study, a multi-center randomized clinical trial. Longitudinal relationships between cognitive domains (i.e., memory, attention, processing speed, executive function) and accelerometer-measured change in daily minutes of physical activity were examined using multivariable linear regression. Cognitive tests included Modified Mini-Mental State Examination (3MSE), Hopkins Verbal Learning Test (HVLT), Digital Symbol Substitution Test (DSST), Task Switching Test, Eriksen Flanker Test and N-Back Test. Physical activity was measured longitudinally using an accelerometer and was categorized to total, light, and moderate to vigorous according to standardized activity count cut points.

**Results:**

Our results showed an association between cognitive change in the processing speed domain and change in minutes of total (β = 0.47; 95% CI: 0.04, 0.89; p = 0.034) and light (β = 0.42; 95%CI: 0.03, 0.80; p= 0.033) physical activity. Relationships between all other cognitive assessments and physical activity categories were not statistically significant.

**Conclusions:**

This study demonstrates an association between a decline in processing speed and a decline in the duration of total and light daily physical activity in older adults with low to moderate physical function.

